# 

**Published:** 1888-10

**Authors:** 


OBSERVATIONS UPON THE MORPHOLOGY OF GALLUS BANKIVA OF INDIA. R. W. Shufeldt, M.D., C.M.Z.S.... 343
ORIGIN OF THE DOMESTICATION OF THE HORSE. Rush Shippen Huidekoper, M.D.. ..... 377
A COMMON LESION OF THE HORSE. Daniel D. Lee, M.D.V...... 389
ONTARIO VETERINARY COLLEGE, TORONTO, CANADA.... 392
EDITORIAL DEPARTMENT.... 397
CASE DEPARTMENT.... 405
PROCEEDINGS OF VETERINARY SOCIETIES.... 415
SELECTIONS FROM FOREIGN JOURNALS.... 427
REVIEWS.... 435
BOOKS AND PAMPHLETS RECEIVED.... 437

